# How to Surveil Perianal Paget's Disease: A Case Report

**DOI:** 10.7759/cureus.50282

**Published:** 2023-12-10

**Authors:** Meryem A Al-Abid, Cameron Law, Isabella Mor

**Affiliations:** 1 General Surgery, Royal Brisbane and Women's Hospital, Brisbane, AUS; 2 General Surgery, The Tweed Hospital, Gold Coast, AUS; 3 Colorectal Surgery, John Flynn Private Hospital, Gold Coast, AUS; 4 Colorectal Surgery, The Tweed Hospital, Gold Coast, AUS

**Keywords:** perianal paget’s disease, extramammary paget’s disease, incidence and prognosis, colon cancer surveillance, colorectal cancer

## Abstract

Perianal Paget’s disease (PPD) is a rare manifestation of extramammary Paget’s disease. It is characterized by the presence of malignant glandular epithelial cells within the squamous epithelium. There is a well-established but poorly understood association between PPD and underlying malignancy. Due to the rarity of the disease, there are no established guidelines for treatment or surveillance of PPD. We present the unusual case of a 73-year-old woman with primary PPD without an underlying malignant lesion.

The rarity of the disease renders its management and surveillance an ongoing challenge. Our case of PPD without an underlying malignancy poses the question of the most appropriate surveillance for this rare disease.

## Introduction

Perianal Paget’s disease (PPD), initially described by Darier in 1893, represents a distinct manifestation of extramammary Paget’s disease (EMPD). The earliest record of Paget’s disease can be traced back to 1874 when Sir James Paget first documented its occurrence in patients with breast cancer [1-3]. It is characterized by the presence of malignant glandular epithelial cells (Paget’s cells) within the squamous epithelium. EMPD is found in apocrine glandular epithelial tissues including the vulva, penis, perineum, perianal, and axilla [4].

PPD is thought to account for less than 6% of Paget’s disease cases. It can be classified as primary, of cutaneous origin; or secondary, result of Pagetoid spread from anorectal or urogenital adenocarcinomas. The pagetoid spread refers to the proliferation of individual neoplastic cells upward into the epidermis, which is typically characterized by erythema and inflammation [1,5].

The association between PPD (both primary and secondary) and underlying malignancy is well-established but poorly understood. The rate of associated malignancy with PPD ranges from 33% to 86% [4,6]. The finding of PPD should prompt a physician to undertake a diligent search for an underlying malignancy.

With less than 200 cases reported in the literature, the rarity of the disease means there are no established guidelines on the treatment and subsequent surveillance [1]. The mainstay of treatment is surgical resection; however, recurrence is common even with negative margins [2].

We present the unusual case of a 73-year-old woman with primary PPD without an underlying malignant lesion.

## Case presentation

A 73-year-old female was referred to our colorectal unit with an irritating perianal lesion present for several months, refractory to topical remedies, with a punch biopsy performed in the community revealing EMPD. 

The patient’s medical history was notable for Hashimoto’s disease, chronic fatigue syndrome, and anxiety. A colonoscopy one year prior had not revealed any polyps or colorectal lesions.

**Figure 1:**
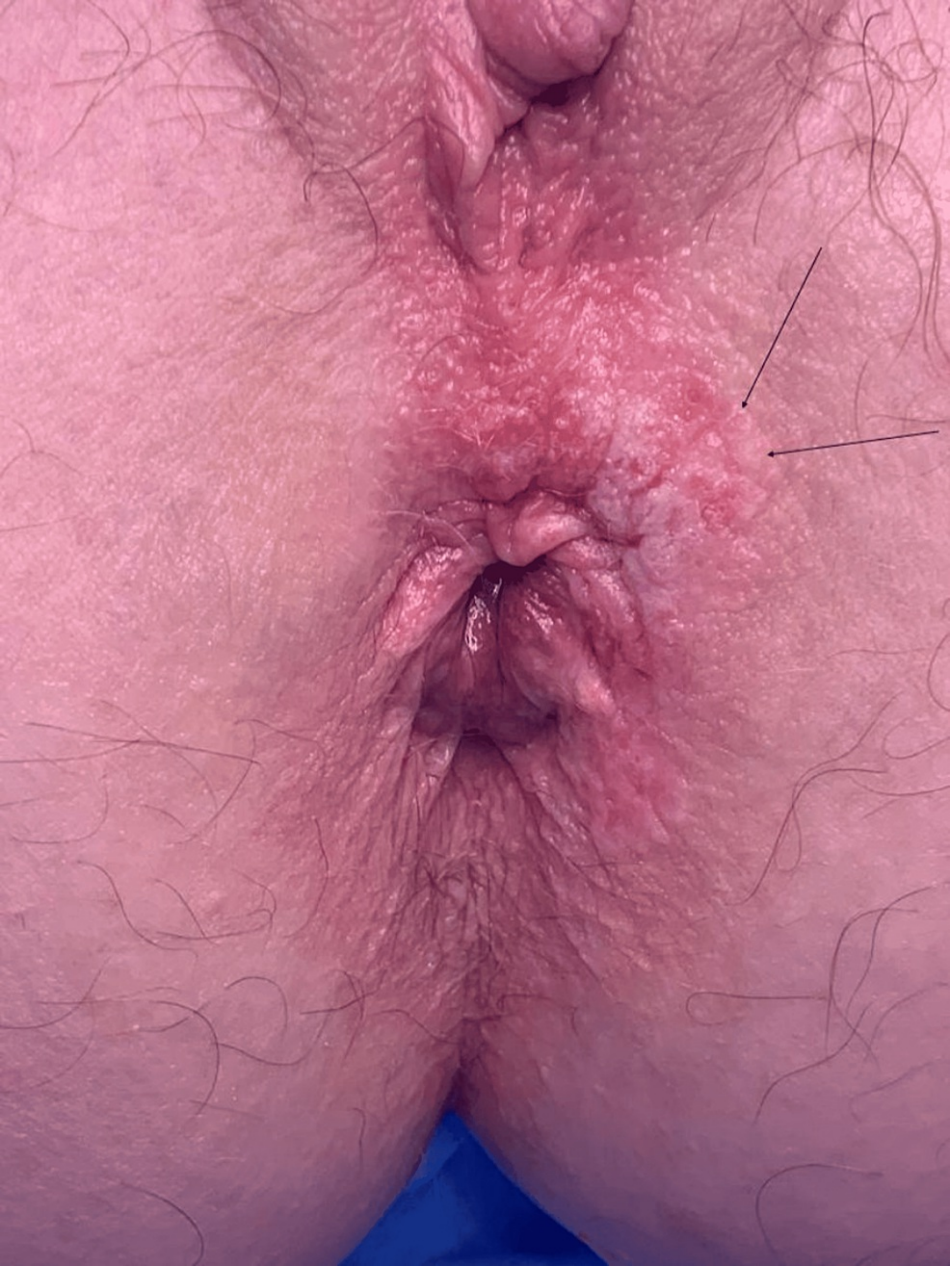
Image of the perianal lesion

She underwent a flexible sigmoidoscopy, which excluded any anorectal or distal colonic lesions. Examination under anesthetic (EUA) revealed a 1 cm eczematous plaque anterolaterally extending from the anal verge (Figure 1). This was excised with a 0.5 cm to 1 cm macroscopic margin. Histology from the excision confirmed PPD without invasion of the basement membrane, unfortunately with a positive surgical margin. Following this procedure, she had staging CT scans, which were negative for local or distant malignancy.

A multidisciplinary discussion recommended re-excision for clear margins, though if the area is deemed too wide for adequate clearance, alternative therapy should be considered. A second wide excision with 1 cm margins occurred two weeks later, at which point it was noted there was a macroscopic extension of the eczematous plaque. Unfortunately, histology from the re-excision still demonstrated involved margins. Due to the disease’s extent, and concerns regarding potential deformity from further surgery, local radiotherapy was recommended.

Having concluded a four-week course of radiotherapy, she underwent a subsequent EUA, which revealed no discernible macroscopic disease. We have now advocated for her to undergo regular surveillance with the colorectal unit.

## Discussion

PPD is a rare form of EMPD accounting for less than 6% of EMPD [1,2]. It has a well-established but poorly understood association with underlying malignancy. Primary and secondary PPD are thought to be different entities, with primary PPD arising as a primary cutaneous lesion while secondary PPD is associated with a colorectal malignancy. Nevertheless, the precise pathogenesis remains incompletely understood, as certain studies now suggest a potential association between primary PPD and an underlying malignancy [1,3]. As such the distinction between primary and secondary disease is not clearly established, with no reliable immunohistochemical difference between the two [1,5]. This is further supported by Wang et al. case series demonstrating that up to 30% of patients with primary PPD will develop metachronous malignancies. Hutchings et al. also report a number of cases that affirm there may be an alternate mechanism to account for the association between PPD and malignancy.

Given the rarity of the disease and the heterogeneity among the reported cases, there are no established guidelines for treatment. Surgical resection is the primary recommended therapy for an appropriate surgical candidate [2,3,7]. This ranges from wide local excision (WLE) to abdominoperineal resection depending on the extent of the lesion and the presence of colorectal malignancy. Although there is no consensus regarding the optimal surgical procedure, in the absence of malignancy, WLE is preferred. Resection margins of up to 3 cm have been proposed; however, this leads to considerable tissue loss, often requiring reconstruction with local advancement and rotational flaps and in some cases a temporary colostomy [8]. Frozen section analysis for margins has been proposed to reduce the recurrence rate; however, negative margins have not been shown to predict local recurrence [4,9]. Nonsurgical treatment options include topical therapies, radiotherapy, and chemotherapy. The role of these therapies remains uncertain and systemic chemotherapy as mono-therapy is considered in patients who are unfit for surgical intervention or those who have widespread multifocal disease [2,8]. Radiotherapy and topical therapies can be utilized as peri-operative adjuncts [2].

In addition to the association with malignancy, it has been shown that recurrence rates of PPD after resection are as high as 60% [1,4]. This prompts the need for close surveillance; however, as with the paucity of treatment protocols, there are no established surveillance guidelines [3,8]. Most recommendations are based on anecdotal evidence and are contingent on the preferences of individual practitioners or clinical centers. These may encompass a range of approaches, including physical examinations, biopsies, anoscopy, and colonoscopy. Rudnicki et al. propose an extensive surveillance protocol with physical examination and anoscopy every six months for the first two years, extended to yearly if there is no recurrence at two years. As well as a colonoscopy at one-year post-resection and if normal, repeat colonoscopy at three and five years.

PPD is an important consideration in patients with perianal itching or a perianal eczematous lesion, particularly due to its association with underlying malignancy. We present a rare case of PPD where no associated malignancy was identified. The case was successfully managed through a combination of surgical excision and radiotherapy. However, even with the successful clearance of PPD, the pertinent question remains regarding the optimal surveillance strategy, considering the acknowledged high risk of recurrence and the potential for the development of colorectal malignancy.

## Conclusions

PPD is a rare subset of EMPD with a poorly understood but well-established association with malignancy. With a nonspecific clinical presentation, it remains an important diagnostic consideration in a patient with perianal itching and perianal erythematous or eczematous changes. The rarity of the disease renders its management and surveillance an ongoing challenge. Our case of PPD without an underlying malignancy poses the question of the most appropriate surveillance for this rare disease.

## References

[1] Wang YC, Li AF, Yang SH, Ma HH, Liang WY (2019). Perianal Paget’s disease: the 17-year-experience of a single institution in Taiwan. Gastroenterol Res Pract.

[2] Thompson HM, Kim JK (2021). Perianal Paget's disease. Dis Colon Rectum.

[3] Rudnicki Y, Stapleton SM, Batra R, Gan T, Mathis KL, Kelley SR (2023). Perianal Paget's—an aggressive disease. Colorectal Dis.

[4] Imaizumi J, Moritani K, Takamizawa Y, Inoue M, Tsukamoto S, Kanemitsu Y (2023). A review of 14 cases of perianal Paget's disease: characteristics of anorectal cancer with pagetoid spread. World J Surg Oncol.

[5] Hutchings D, Windon A, Assarzadegan N, Salimian KJ, Voltaggio L, Montgomery EA (2021). Perianal Paget's disease as spread from non-invasive colorectal adenomas. Histopathology.

[6] Liao X, Liu X, Fan X, Lai J, Zhang D (2020). Perianal Paget's disease: a clinicopathological and immunohistochemical study of 13 cases. Diagn Pathol.

[7] Santos MD, Soares F, Presa-Fernandes JM, Silva DS (2021). Perianal Paget disease: different entities with the same name. Cureus.

[8] Rajendran S, Koh CE, Solomon MJ (2017). Extramammary Paget's disease of the perianal region: a 20-year experience. ANZ J Surg.

[9] Isik O, Aytac E, Brainard J, Valente MA, Abbas MA, Gorgun E (2016). Perianal Paget's disease: three decades experience of a single institution. Int J Colorectal Dis.

